# ‘Playing war’: Norwegian soldiers’ experiences of fun and responsibility in Afghanistan

**DOI:** 10.1080/07292473.2024.2409539

**Published:** 2024-10-14

**Authors:** Heidi Mogstad

**Affiliations:** Chr. Michelsen Institute, Postdoctoral Researcher, Bergen, Norway

**Keywords:** soldiering, fun, responsibility, Norway, Afghanistan

## Abstract

This article discusses Norwegian soldiers’ experiences of fun in Afghanistan and probes the meaning of this experience and its condition of possibility. Challenging Western moralities of war, it shows that ‘fun in war’ can be experienced and represented as ordinary and commonsensical, but also ephemeral and immature. Moving beyond normative and functionalist approaches, it specifically highlights the importance of context, temporality, and soldiers’ sense of responsibility and innocence.

In popular and scholarly imaginations, war and fun are usually considered antithetical. This was brought home to me many times over the past year when I told friends, family, and colleagues about the new research project I was part of, entitled WARFUN. While many found the title of the project provocative or inappropriate (especially in the context of Russia’s full-scale invasion of Ukraine, which began only a few weeks after I embarked on the project in February 2022), others questioned the value of the research or said they struggled to understand how anyone can experience war as fun. The lived experiences of soldiers, however, often differ radically from public narratives and understandings.^1^ Moreover, as ethnographers and scholars, we should be careful not to smuggle our own morality of war into the analysis, thus foreclosing other interpretations and moralities.

This article discusses Norwegian soldiers’ experiences of fun in Afghanistan and probes the meaning of this experience and its condition of possibility. Drawing on in-depth semi-structured interviews with current and former soldiers in the Norwegian Armed Forces, it shows that ‘fun in war’ can be experienced and represented as ordinary and uncontroversial, but also as transgressive, ephemeral, and immature. Moving beyond normative and functionalist approaches, it specifically highlights the importance of context, temporality, and soldiers’ sense of responsibility and innocence.

The article proceeds as follows. I begin by discussing what fun is by turning to the pioneering work of sociologist Ben Fincham. I thereafter elaborate on and qualify the idea of war and fun as antithetical by suggesting that some forms and expressions of fun in war are seen as acceptable, while others are coded as sick and deviant. Next, I introduce my methodology and sample, before presenting war stories from Norwegian Afghanistan veterans that challenge this distinction and unsettle Western moralities of war as tragic and regrettable. Finally, I try to make sense of my interlocutors’ experiences of fun in Afghanistan in conversation with Fincham’s ‘peacetime theory’. I also discuss when war is no longer experienced as fun, thus bringing temporality and change into the analysis.

## What is fun?

Before I discuss Norwegian soldiers’ experiences of fun in Afghanistan, it is important to establish what fun is and how it differs from other emotions and experiences. This is easier said than done, however, since fun is usually conflated with, or treated as an outcome or by-product of, other sensations considered more susceptible to theorising, such as humour, enjoyment, happiness, or pleasure.^2^ Moreover, fun is ‘curiously ambiguous – we know when we are having it, but struggle to define it’.^3^

While some efforts have been made to theorise fun in psychology,^4^ leisure studies,^5^ and computer science,^6^ Fincham’s book is the first comprehensive attempt to theorise fun from a sociological perspective. It traces the word’s etymology but relies predominantly on data from a qualitative online survey conducted in 2014 with mostly middle-class British people of both genders.^7^ Despite some limitations with this methodology (and the obvious difference that Fincham’s respondents are British civilians in peacetime), his model of fun provides a useful starting point for my analysis.

While recognising that there are clear overlaps between fun and other affective states and sensations, Fincham argues that fun has some distinct qualities that help us understand its role and importance in people’s lives. To begin with, Fincham argues that fun is characterised by its fleeting and tangible nature. Fun does not ‘resonate or echo in time’ the way that happiness and pleasure can but is usually transient and short-lived.^8^ Compared with enjoyment and pleasure, fun also tends to have more specific and everyday meanings.^9^ Like play, experiences of fun can be located in time and place and often have clear beginnings and ends.^10^

Moreover, for ‘something to be fun, there needs to be an absence of seriousness’.^11^ An embodied sensation, fun is associated with lightness, cheerfulness, and laughter.^12^ Although increasingly recognised as healthy and vital for our wellbeing, experiences of fun are thus commonly seen as marginal or trivial and typically associated with time wasting, unsophistication, and childishness.^13^ Additionally, fun can be characterised by what it does to us. When we have fun, we are distracted from our everyday lives and problems and become completely immersed in our activities.^14^ Importantly for the argument I will make later, fun also involves an at least temporary alleviation of commitments and responsibility. As Fincham specifies, it is not that fun is defined through irresponsibility, but that responsibility is not a concern during periods of fun.^15^

Four more points should be highlighted. First, like other social phenomena, fun is contextual and mediated by culture, history, and society.^16^ Second, fun is social and interactional. Unlike happiness and pleasure, which can be experienced individually and alone, fun is usually had with other people or at least in relation to other people, as experiences of fun require affirmation from others.^17^ Moreover, unlike humour, fun moments are non-hierarchical or involve a disregard or negation of power differentials.^18^ Third, fun is a multi-layered phenomenon with a complex temporality. As indicated, it is experienced in the moment and the body. Fun is also a discourse, which is applied retrospectively.^19^ Indeed, many events and experiences are reconstructed or retold as fun, despite not being felt as such in the moment.^20^ Analysing this seeming paradox, Fincham notes that memories are shaped by social norms and present concerns, including the need for meaning and coherence. Moreover, stories of fun are closely related to our personal and social identity – who we think we are and how we like to be seen by others.^21^

Fourth and finally, fun has an ambiguous relationship to power. On the one hand, our conceptions and experiences of fun are influenced by hegemonic norms and discourses and the commodification of fun in the leisure industry. On the other hand, Fincham observes that there is very often a transgressive element to how we have fun.^22^ As he specifies, ‘transgressions need not be some sort of grand statement; it may simply mean not really doing what you are supposed to or what you normally do’.^23^ Considering the possibility of having fun at work, Fincham suggests that the sort of fun workers have stands in contrast to the ideal of a good worker and institutional control of time and behaviour.^24^

To sum up, Fincham defines fun as a fleeting and frivolous experience which, like play, often has a clear beginning and end. He further analyses fun as a distraction from everyday life and routines and argues that it involves an absence or temporary lessening of personal commitments and responsibilities. Fun has, however, a complex temporality as it is also a discourse applied post hoc that is important for people’s identity. Finally, fun is contextual, social, and often experienced as transgressive.

## Fun in war

Given fun’s association with play, lack of seriousness, and transgression, it is perhaps easy to understand why many find the idea of having fun in war surprising or inappropriate. According to Western moralities, war is a serious and tragic event.^25^ Whether we see a particular war as morally just and necessary or completely senseless, we know war by the pain and suffering it inflicts on people and societies. To paraphrase Hemingway,^26^ we know war is bad, and anyone who says it is not, is a liar. Moreover, being a soldier in war is usually framed as a duty and a sacrifice.^27^ According to mainstream narratives, soldiers return home from war traumatised and alienated from society.^28^ They are expected to suffer from post-traumatic stress disorder (PTSD) or moral injury,^29^ not to speak about how much fun they had.

To associate war-making with fun might be particularly controversial in some countries and military cultures. For instance, Eva Johais in this special issue argues that there is a strong taboo against naming war as fun in German society given the country’s violent past and anti-militarist social climate.^30^ Occupied by Germany during the Second World War and self-proclaimed outsiders of the European colonial project, Norway has a more glorified and sanitised national history defined by heroic resistance and innocence.^31^ A major donor of aid and development assistance, Norway also has a self-congratulatory public self-image as a ‘moral and humanitarian superpower’.^32^ In accordance with this self-definition, Norwegian soldiers in Afghanistan were publicly framed as non-violent peacemakers, building schools and wells in Afghanistan.^33^ Moreover, Norwegian military leaders and scholars regularly describe Norwegian soldiers as exceptionally well behaved and ethical, suggesting that they are more sensible and restrictive with violence than other national armies.^34^

In the light of this Norwegian exceptionalism, the idea that Norwegian soldiers were having fun in Afghanistan, and especially having fun while engaging in combat, is difficult to digest for a public used to thinking of their soldiers in heroic and innocent terms. It also sits uneasily with the media coverage of the war and Afghanistan veterans, which focused overwhelmingly on Norwegian casualties and injuries.^35^

Notably, there are some exceptions to what Johais describes as a ‘WARFUN taboo’: a widespread societal repulsion against associating war with fun. For instance, it is usually tolerated that soldiers have fun when off duty or inside the base, while ‘waiting for war’^36^ or participating in organised forms of fun such as entertainment and recreational activities or holiday celebrations.^37^ In such settings, spatially and temporally removed from the battlefield, it is understood that soldiers, like everyone else, need to have fun to stay healthy and sane. Moreover, dark humour or gallows humour is an expected and acceptable feature of soldiering and typically interpreted as a stress release or coping mechanism. Two points should be made about these acceptable forms of fun. First, they are usually said to have an important function, whether consoling soldiers or promoting bonding and unit cohesion.^38^ Second, the widespread tendency to interpret fun in war as a way to escape or cope rests upon and reinforces the idea that war is ultimately experienced as tragic and agonising. Arguably, these forms of fun are tolerable because they confirm ‘our’ moralities of war and what it means to be a soldier.

Other expressions of fun in war tend to be treated as deviant and out of place, associated not only with disobedience or indiscipline,^39^ but also with wrongdoing and immorality. Consider, for instance, the notorious Abu Ghraib abuses, where convicted US soldier Lynndie England explained that the abuse of Iraqi war prisoners was not only about interrogation and domination in the name of state power but also ‘just for fun’.^40^ As John Parry observes, many commentators believed this comment demonstrated the exceptional nature of the abuse, thus drawing a clear line between a few soldiers’ personal misconduct – condemned as perverse, disgusting, and immoral – and the ‘proper, upright conduct of the normal state actor’.^41^

Another representation of fun in war as a deviant and ‘sick’ affect is found in the book *Den Første Sten* (*The first stone*) by the Danish writer and public intellectual Carsten Jensen.^42^ The book is fictional but hailed as a powerful and important work about the war in Afghanistan. Crudely summarised, *Den Første Sten* follows a group of young Danish soldiers on the ground in Helmand province. The Danish soldiers had voluntarily signed up to go to Afghanistan and were motivated by a desire to experience war and test their skills and courage. Confronted with the moral chaos of the war and the personal betrayal of their platoon leader, however, their sense of right and wrong is called into question and they became obsessed with revenge. The book concludes with the few surviving Danish soldiers crying, not only over the deaths of their comrades but in response to everything they had seen and done.

As alluded to in the title, the book thus illustrates the extreme brutality and senselessness of the war in Afghanistan as well as the moral corruption of war in general. The most provocative element, however, is not the words and actions of the Danish soldiers, but those of their charismatic platoon leader Shrøder. While the others become shocked and disoriented by the moral chaos of the war, it turns out that Shrøder thrives in this environment. Self-described as a ‘vagabond’, ‘scoffer’, and ‘unholy warrior’, Shrøder, who we learn is a former video game producer, treats the war as a game. Aided by his cultural knowledge and language skills (Shrøder speaks fluent Pashto), he changes sides opportunistically, playing and betraying not only his Danish comrades and allies but also the Taliban and a group of abusive American private security contractors. When Shrøder is asked by the Danish intelligence agent who comes looking for him about his motivation, he replies: ‘Isn’t it obvious? I am here to have fun. To see how far I can take this.’^43^

Rather than a coping mechanism or bonding activity, this conception of fun is associated not only with play and gaming but also with moral nihilism and destruction. Like the commentators writing about the Abu Ghraib abuses, the book thus portrays fun in war as a deviational and perverse affect that is even more harmful than the Danish soldiers’ lust for revenge and lack of cultural understanding. Is it though so exceptional to have fun in war or to think of the war in Afghanistan as a form of play? Moreover, does fun in war need to involve either stress release or serious misconduct and war crimes?

## Methodology

This article is based on research with Norwegian soldiers and war veterans who served in professionalised expeditionary forces in Afghanistan. Lasting from December 2001 to August 2021, Norway’s mission in Afghanistan is one of the largest, lengthiest, and most costly international operations Norway has participated in since the second world war. Over the course of twenty years, about 9,200 Norwegian regular and special forces soldiers served, mainly in Faryab province in northern Afghanistan, where Norway assumed responsibility for regional stability, but also in Mazar-e-Sharif and Kabul.^44^

While Norway has actively contributed to peacekeeping missions in Lebanon, Africa, and the Balkans, the US and NATO-led mission to Afghanistan differed in many respects. Most importantly, Norway’s contribution to Afghanistan followed a shift in military practice and thinking away from the traditional role of territorial defence and conscription (increasingly viewed as a burden) and towards a more compact and professional army focused on international operations.^45^ Only conscripts who were contracted served in Afghanistan. Hence, Norway sent fully professionalised troops with soldiers who often knew each other and had trained together and prepared themselves well. Moreover, despite publicly framed as a humanitarian intervention, Norway’s contribution to Afghanistan was driven by the country’s commitment to support the US and demonstrate that Norway is a reliable and ‘good NATO ally’.^46^ Compared with earlier peacekeeping missions, Norway’s contribution to Afghanistan was thus viewed as strategically important. It was also considered positive for soldiers’ training and careers.

In Afghanistan, Norwegian citizens participated regularly in combat for the first time since the resistance against Nazi Germany’s occupation of Norway between 1940 and 1945.^47^ Between 2004 and 2011, ten Norwegian soldiers were killed and several more were wounded. While these losses cannot be compared with the costs of war to the Afghan population,^48^ or the considerably higher losses of many allied forces, the deaths of Norwegian soldiers became public events at home and prompted the army to prioritise the security of their own personnel further.^49^ Nevertheless, Norwegian troops remained in Afghanistan until the summer of 2021, when NATO withdrew all Allied personnel, paving the way for the Taliban to return to power.^50^

To explore Norwegian soldiers’ experiences of the war in Afghanistan, I used a variety of methods, including media and document analysis and archival research. I also visited and participated in events at military camps and academies, veteran centres, and military museums. My primary data collection method, however, was in-depth, semi-structured, and occasionally repeated interviews with current and former soldiers in the Norwegian Armed Forces. My interviewees were mainly men (twenty-two men vs six women) aged thirty to fifty-eight, but they had different class and educational backgrounds and came from all regions of Norway.

Due to my interest in soldiers’ relationships to violence and enemy construction, I purposefully sought out people with combat experience. Apart from this criterion, I interviewed soldiers with different ranks, military specialities, and careers, ranging from special forces operatives and intelligence officers to infantry and other enlisted personnel. The rationale behind this diversity was partly to get a varied picture of Norwegian soldiers’ views and experiences but also to identify common tropes and narratives that cut across rank, function, and unit culture. Significantly, Norway has conscription ingrained in its constitution and a long tradition of recruiting soldiers from all walks of life to create a ‘people’s army’.^51^ Partly because of this, but also due to Norway’s welfare system and egalitarianism, the military is not usually seen as a pathway to socio-economic mobility or citizenship like it is in many other countries. The aforementioned shift to a more compact and professionalised army has though led the army to implement conscription more selectively, focusing on recruiting ‘suitable’ and motivated youths.

For many of my interviewees, serving abroad was a key motivation to seek a career in the army. Like the Danish soldiers in Jensen’s book, the vast majority thus signed up to go to Afghanistan voluntarily and were motivated primarily by a desire to experience war and test themselves under difficult circumstances.^52^ A few also said they had initially hoped to ‘help Afghans’ or ‘fight terrorism’, though such humanitarian and ideological motivations were always secondary, and most said they quickly lost faith in the mission.

Their tours lasted between three and nine (usually six) months, and many returned on several missions. As indicated, most interviewees had experienced combat and two had received individual decorations for their acts of valour. Several had lost friends or colleagues in Afghanistan, two were still suffering from war-related injuries, and two had been diagnosed with PTSD. Nevertheless, the vast majority said they had grown as a result of the experience and had become more satisfied and grateful. This corresponds with the most recent ‘Afghanistan survey’, which found that one out of ten veterans suffers from psychological problems, while 79 per cent of the respondents reported good life quality.^53^ About half of my interviewees were still working for the army during our interview, and some had recently served in Lithuania or Syria. Others had transitioned into civilian careers but typically worked in related professions as police officers, firefighters, or emergency health workers.

Interviewees were mainly recruited *via* personal networks and the snowball method, but I also contacted some *via* email and social media. While I come from a fairly anti-militarist family and have never had any desire to become a soldier or experience warfare, my partner works for the Armed Forces and together we have many friends and acquaintances who have served in Afghanistan. When approaching and speaking to soldiers, I found honesty about both these aspects of my position to be ethical and helpful to building trust, while also allowing me to ask ‘stupid’ and critical questions.^54^

The interviews were conducted in various spaces according to the soldiers’ preferences, including their homes or workplaces, quiet cafés, or outdoors. They lasted between one and three hours and were typically informal and conversational. All interviews were conducted in Norwegian, and the majority were recorded. While my research focuses specifically on fun and other ‘positive’ sensations, I always began interviews with broad, open-ended questions to see what words the soldiers chose to narrate their experiences. The interviews also probed soldiers’ difficult and ambivalent experiences and questions of meaning and responsibility.

Notably, there are two Norwegian words for fun*: gøy* and *moro*. While both words were used in interviews and conversations, I mainly used *gøy* when asking questions. According to the Norwegian Academy’s dictionary, the word’s etymology is unknown but likely stems from the word *ablegøye* and the Dutch *babbelguigje*, which can be roughly translated as prank or mischief. *Gøy* is predominantly used orally and has strong connotations of childhood, carefreeness, and play, like the English equivalent.^55^ In everyday speech, the word is commonly used to describe activities that a person enjoys doing, for example a sport or social activity, but it can also be used to describe a happening or event. The second word, *moro*, is used similarly but is more closely associated with humour and entertainment.

The recorded interviews were transcribed and analysed alongside fieldwork notes using thematic, narrative, and discourse analyses. Selected quotations have been translated into English by the author. This article foregrounds the experiences of three anonymised soldiers. To ensure confidentiality and minimise the risk of misinterpretation, names are anonymised, and some details have been altered or omitted based on dialogue with the three individuals. Nonetheless, the analysis is based on my scholarly interpretation, and I do not claim to speak on behalf of my interviewees or to have understood everything they told me.^56^ Recognising the ethical complexities of conducting research on ‘things military’, I consider my research an empathic but critical interrogation of military lifeworlds and frames of reference and take full responsibility for any errors, misinterpretations, or other shortcomings.^57^

## Norwegian soldiers’ experience of fun in Afghanistan

I began this article by describing some of the reactions I got from friends and colleagues when telling them about the WARFUN project. Whether shocked, provoked, or simply surprised, they found the association between war and fun curious or troubling. The Norwegian soldiers and veterans I interviewed displayed different reactions. While a few described the title as ‘a bit sensationalistic’ or ‘tabloid’, the majority nodded affirmatively when I introduced the project. The idea that soldiers were having fun in war was neither shocking nor offensive to them but quite trivial and commonsensical. Several also said they appreciated the project’s focus or angle and that this was part of why they had agreed to talk with me. Stories about war veterans in Norwegian media focus overwhelmingly on their struggles or traumas, contributing to what several interviewees described as ‘negative stereotypes’ or ‘stigmas’. Conversely, the framing of the WARFUN project resonated with them and ‘indicated that I was interested in listening to their full experience’, as one veteran put it.

Certainly, the soldiers I interviewed were firmly aware that civilians in Norway might find the association between war and fun (*gøy)* problematic. Consequently, many said they would not use this word to describe their experience in Afghanistan in public or to civilian friends and family members. Some also said they did not feel that fun was the most accurate word to capture their experiences and preferred related adjectives such as thrilling, interesting, or enjoyable. Many others though used *gøy* or *moro* explicitly and sometimes unsolicited in our conversations. Besides pointing to specific moments or episodes, several also described their entire mission in Afghanistan as *gøy*.

In what follows, I foreground the experiences of three Norwegian soldiers with different roles and careers in the army. While not representative in a statistical sense, these cases are selected to illustrate my diverse sample and common experiences and representations of fun in my material. While focusing on the personal experiences of three individuals gives texture and helps to substantiate my arguments, I also analyse these cases in relation to other interviews and literature.

### Simon

Simon served on a Military Observatory Team in northern Afghanistan for seven months in 2008 and 2009. Born and raised in an affluent suburb of the Norwegian capital, he had always been fascinated by war and weapons. Like most of the male soldiers I interviewed, he grew up playing war in the forest with his childhood friends, and as a teenager, he eagerly waited for his US fighter pilot magazine to arrive in the mail. After completing his military service, he started studying at the university but found civilian life dull and disappointing. Simon was twenty-three years old and had recently finished a bachelor’s degree in economics when he went on his first and only tour to Afghanistan. To illustrate how he talked about his experience, I quote from our interview:

**Me:** You said earlier you wanted to go to Afghanistan ‘because of the experience’. Would you describe this experience as fun?

**Simon:** I thought it was a lot of fun!

**Me:** What was fun about it?

**Simon:** We were basically tourists there. We just travelled around on our own. I mean, not really tourists, because we had a job to do. But we had a big operational area and travelled around so our liaison could speak to all kinds of people: the police, local chiefs, Afghan military, village leaders, border guards, NGO [non-governmental organisation] personnel … A typical day could entail visiting villages, an NGO project, and a checkpoint or a border, patrolling and showing our face here and there and checking out this and that. And then, we usually had a break in one of the local villages, where we bought soda and bread to charge our batteries and spread some dollars and goodwill. Things like that. We often planned our missions and spent many days outside the base, camping in nature on beautiful hills with amazing views. We also travelled through small, quirky villages and beautiful landscapes. And we often got to speak with local people and kids. It was really a lot like backpacking!

The idea of soldiering as an opportunity to travel and see the world is far from new and carries a problematic legacy of colonial adventurism and self-realisation.^58^ What I wish to highlight here, however, is how Simon described his ordinary soldiering tasks as fun and associated this with civilian activities he also enjoys, namely camping outdoors and travelling or backpacking. Surely, not every moment in Afghanistan was fun or exciting. In our interview, Simon recollected that he was frequently bored, wet, and cold:

**Simon:** It is incredibly boring most of the time, so you must try to enjoy what you see and experience. The nature, villages and culture. Drinking tea and talking to locals. Being there was truly joyful, but it was not like a *sydenferie* [charter tour to Southern Europe]. It got really cold; at one point, it was minus twenty degrees and on a different assignment, we had a whole week of rain, zero degrees, and sleet. And I was always sitting in an open car. During my seven months in Afghanistan, I was not once inside the car or behind a window. Hence, I was frequently cold and wet and must admit I got really fed up sometimes.

**Me:** I would have hated freezing for so long! But despite this, you describe the mission as mostly fun?

**Simon:** Yes, for me, the whole experience was overwhelmingly positive. I had a really good time, and I also got to test myself and my skills and felt a lot of mastery (*mestring*).

Captured by the popular expression ‘hurry up and wait’, boredom has long been recognised as an integral part of soldiering life.^59^ The Afghanistan veterans I interviewed often suggested that soldering consisted of one per cent action and 99 per cent waiting or boredom. As exemplified by Simon’s comment, many interviewees also recalled being wet and cold or tired and frustrated with their superiors, military rules, or their limited mandate and progress on the ground. Despite such pains and frustrations, though, interviewees described their tour(s) in Afghanistan in affirmative terms. While generally happy with their current lives and jobs, many also said they missed their time in Afghanistan, associating it with cheerfulness, lightness, camaraderie, and adventure. As Simon replied, when I asked him to compare his deployment in Afghanistan with his current job in the Norwegian police force:

I feel more useful in my current job since I have done things that make a positive difference for society […]. However, I like travelling and nothing beats the tingling sense of excitement that anything can happen at any time.

As this comment indicates, being a soldier in Afghanistan might involve pleasurable ‘civilian activities’ such as camping and travelling, but the context of war added to these experiences by creating an alluring atmosphere of thrill and uncertainty. It is also worth highlighting that Simon and many other interviewees described Afghanistan as a significant but closed chapter in their lives. Moreover, they remembered this chapter as personally meaningful but not necessarily ‘useful’ for the wider society. I return to these points below.

### Katrine

Born in southern Norway, Katrine said it was coincidental that she ended up in the army. She had always been an athletic and energetic girl who enjoyed being outdoors, climbing and playing football, but at the time Katrine graduated, military service was not compulsory for women in Norway. Moreover, after high school, Katrine’s initial plan was to take a year off and go backpacking in Asia. She had, however, an older brother who worked for the Norwegian Army, who convinced her to apply to their language school. After a year of intense language study, Katrine was sent to Kosovo, where she served as a combat interpreter. ‘Being allowed to do this job at the age of twenty was incredibly fun’, she recalled in our interview.

Katrine spent more than ten years in the Armed Forces and had several deployments in the Middle East and Balkans. She was sent to Afghanistan twice, where she served as an area specialist embedded with combat troops. While training at home, including ‘lots of shooting drills and field exercises’, was enjoyable, Katrine especially valued her overseas deployments. Like Simon, she highlighted the joy of travelling and exploring. While Simon likened his deployment to backpacking, Katrine emphasised the uniqueness of her war experiences and compared these with the lives and jobs of her civilian peers in Norway. She also talked about how much she enjoyed serving as an expert:

I loved working under pressure, making quick decisions and being tested. What road is safest to use? Where are we most likely to get attacked? [In Afghanistan] everything I did had direct and sometimes immediate consequences. I thought that was very challenging and very fun!

As illustrated by this quote, Katrine’s experiences of having fun in war involved some serious and potentially deadly responsibilities. Challenging the notion of fun as frivolous and trivial,^60^ she also felt that the fun things she did in Afghanistan were much more important than what her friends and peers were doing at home. Like Simon, she further linked her fun experiences to mastery, specifically the mastery of her job in demanding circumstances.

Again, it is important to highlight that fun was not the only sensation Katrine experienced in war. While Simon spoke of how fun coexisted with boredom, Katrine described several episodes which had been frightening and stressful. Nevertheless, these serious incidents did not prevent her from having fun. As she explained concerning a particularly violent episode: ‘After the incident, we felt the gravity of the situation. But we did not stop having fun together. We were always joking around.’ While this quotatiom might suggest that fun served as a form of stress release and a coping mechanism, Katrine underscored that ‘being there’ and ‘fully experiencing the physical and emotional intensity of her profession’ was something she desired and savoured. Comparing this lust with her current job as a paramedic, she said:

If you are a soldier in Afghanistan, you want to feel that you were there when it happens. That’s what’s fun! It’s the same in my current profession: the crazier and more urgent, the more fun!

### Peter

Peter grew up on a farm in western Norway as the oldest of four children. His grandfather had been part of the resistance against the German occupation during the Second World War, and Peter knew he had blown up train lines and used to hide weapons on the farm. He used to tell Peter he should be grateful for not having to experience a war. Peter felt differently. For as long as he could remember, there had been two things he wanted to do with his life: travel the world and be a soldier. A career in the army enabled him to satisfy both desires. Trained as an artillery hunter, Peter was in Afghanistan four times and faced difficult combat situations, including improvised explosive device (IED) attacks and ambushes. When I asked about these experiences, he said he ‘liked being challenged and thrown into situations where you don’t really know what is happening’.

The soldiers I interviewed had different relationships to risk and insecurity. While Peter and a few others said they thrived in situations of chaos and uncertainty, the majority said they only had fun if they felt ‘a sense of control’. Feeling in control is perhaps not something many of us associate with being at war. Nonetheless, the Norwegian soldiers I interviewed explained that they usually or always felt in control because of their ‘technological and military superiority’ vis-à-vis the Taliban, high trust in their own skills and fellow soldiers, and ability to call on US air support or other reinforcements. Like Katrine, many also emphasised that they had voluntarily chosen to experience war and ‘wanted to be there when things happened’ even if it exposed them to some harm or danger. For most interviewees, their deployment in Afghanistan thus represented ‘calculated risk’ or ‘just the right amount of thrill’ (*akkurat passe spennende*), as a seasoned veteran phrased it.

When I asked if combat was fun, some told me it *could* be fun, especially if the enemy was far away and ‘it almost feels like you are shooting in a video game’. Others frowned at the suggestion. A soldier who received an award for his bravery on the battlefield put it like this: ‘If you think combat is fun, you have not been shot at from a close enough distance’. For him and other soldiers I talked to with experience in close combat, this could be characterised as fun post hoc, after they were no longer in the heat of battle, and especially once they had returned to the base and could relax and decompress with their comrades. As Peter explained:

When you are in the midst of a combat situation, it is not so much fun. But afterwards, you feel extremely relieved and holy shit – this was fun! […] Many things we do in the Armed Forces are like that. While you are in the thick of it, it’s fucking unpleasant, but afterwards, you feel great! […] I believe skydivers and climbers feel the same way. It is the kick you get when it goes well, and you are all done.

Whether backpacking, skydiving, or working as a paramedic, interviewees drew frequent analogues between war and civilian life. This makes their feelings and experiences more intelligible to civilians, who many believe are incapable of fully understanding warfare.^61^ It is though worth underscoring that the analogues only work because the Norwegian soldiers could return to a safe base and unwind after a dangerous situation or combat. As I elaborate below, their experiences and feelings are thus shaped by their particular mode of soldiering, including their asymmetric and distant relationship to the Afghans they were tasked to fight or protect.

While making parallels to civilian life, interviewees also described war as a special context which blurs the line between the ordinary and extraordinary and enables a particular form of lifestyle and sociality they experienced as pleasurable and fun.^62^ I quote from my interview with Peter:

If I think about the mission in its entirety, it was fun. We are sitting in a hole in Afghanistan, sharing a Red Bull and having tons of fun together. The jargon, the gallows humour, and the entire lifestyle you have down there – it is actually a lot of fun!

As feminist scholars have shown, the military and war are both constructed as masculine spaces.^63^ Moreover, regulation and display of emotions in the military are clearly gendered.^64^ Affirming this, male interviewees often used the Norwegian term *guttastemning* when describing the type of fun they had in Afghanistan. The term is difficult to translate into English, but according to Urban Dictionary, it describes a euphoric feeling or mood boys experience when they are ‘in their right habitat’. I would add that it usually involves jokes and pranks. What about the female soldiers then? While research on the Norwegian military has argued that women are seen as a threat to masculine brotherhood and cohesion,^65^ the few female soldiers I interviewed emphasised that their gender became *less* relevant when serving abroad and that they experienced *more* inclusion and belonging in Afghanistan. As Katrine indicated, she participated in her unit’s cultural practices and jokes – having fun rather than being made fun of. Significantly, I do not imply that female soldiers in the Norwegian Army were never subjected to marginalisation or harassment in Afghanistan. Moreover, some female interviewees explained that they ‘carefully policed themselves’ to conform with gendered norms or emulate military masculinity. Their experiences of participating in the fun moments and lifestyle in Afghanistan, however, speaks to Fincham’s observation that fun involves negation or neglect of power and other differences.

To understand my interviewees’ experiences of fun in Afghanistan, it is also important to consider their relationship to domestic life in Norway. Research on American shows that domesticity often emerges as an object of normalisation and aspiration.^66^ For my interviewees, life in Norway was likewise associated with normality and affection but also tedious commitments and boredom. Consequently, being deployed in Afghanistan was often framed as a welcome disruption of their everyday life and responsibilities in Norway. As Peter explained:

You don’t have the responsibilities you have at home; you are completely absorbed in the bubble and just concentrate on the tasks ahead of you. You don’t have to drive your kids to school or struggle to get up in the morning to go to work or anything like that.

Nearly all the soldiers I talked to said something analogous to this. Here is another example from a different interview:

Nobody can reach you. And you don’t have any of your usual commitments to your family or other people at home in Norway. You don’t have to worry about bills or think about what you will make for dinner and these sorts of things either.

Significantly, being at war was not framed as an escape from a meaningless or difficult life at home.^67^ Conversely, most interviewees spoke fondly about their domestic work and relationships and said they usually looked forward to coming home after their mission. As seen in the quotations above, being deployed for three to nine months allowed soldiers to become totally immersed in the military ‘bubble’ while simultaneously being distracted or removed from their ordinary responsibilities and routines. Following Fincham, this parallel movement of absorption and distraction is what fun does. In line with Fincham’s theory, interviewees also described fun in Afghanistan as social and interactional – experienced together ‘through and between bodies bounded by gendered and militarized bonds’ and later declared and affirmed by their fellow soldiers.^68^ While seemingly minor and trivial, experiences of fun and camaraderie formed lasting memories which, years later, were still cherished and retold.

## ‘Playing war’

In this article, I have discussed Norwegian soldiers’ and veterans’ experiences of fun in Afghanistan. While we can distinguish fun from other sensations for analytical purposes, their narratives show that fun is related to and overlaps with other experiences and emotions. I have especially highlighted the close link soldiers made between fun and mastery and between fun and thrills, but also how their experience of having fun in war overlapped with feelings of intensity, exploration, adventurism, and camaraderie. The three war experiences I focused on further illustrate how fun coexisted with other, less pleasant sensations such as fear and boredom. In fact, on some occasions, distress and boredom not only coexisted with fun but led soldiers to experience it, whether filling dead time with *guttastemning*, lightening the mood with gallows humour, or applying the status of fun to combat post hoc. Accordingly, we might say that soldiers had fun in Afghanistan not *despite* or ‘in excess of war’ but *because* of it.^69^

Given the widespread societal repulsion against associating war with fun, how should we make sense of these experiences? As discussed earlier, soldiers’ experiences of fun in war are often analysed in functional terms as a coping mechanism or stress release, thus reinforcing Western moralities of war as tragic and regrettable. Having fun is also commonly said to promote bonding and unit cohesion and thus to be instrumental for soldiers’ wellbeing and war efforts. Alternatively, expressions of fun in war that exceed this frame are represented as marginal and deviant and typically associated with immorality and wrongdoing. Moving beyond such normative and functionalist approaches, this article has attended to how Norwegian soldiers narrated their experiences in Afghanistan and the personal biographies and context that defined these experiences. As described, my interviewees generally found the association between fun and war unsurprising and commonsensical and made frequent parallels to civilian life and activities to make this intelligible. Moreover, they spoke of fun as an ordinary sensation which they experienced not only during their leisure time or while waiting for war but while on duty and missions performing their soldierly tasks and responsibilities.

To be sure, I do not suggest that my interviewees’ experiences of fun had no functions or effects on personal or military levels. Conversely, it seems obvious that having fun with their comrades helped to bolster soldierly morale and wellbeing and strengthened military bonds and unity. Some interviewees also told me that ‘joking around’ helped them cope or release stress during or after difficult events such as close combat situations. Moreover, fun emerged in my interviews as a discourse applied post hoc, coloured by time and nostalgia, and likely important for soldiers’ sense of self as adventurers, explorers, and brave, active and out-of-doors Norwegians of both genders.^70^

As demonstrated, fun was not confined to such episodes and purposes. Instead, it was a regular feature of my interviewees’ deployment in Afghanistan and was often used to describe their experience as a whole. Rather than concluding this article with a functionalist interpretation of my interviewees’ experiences of fun in Afghanistan, I will end by analysing its condition of possibility. More specifically, how can Norwegian soldiers and veterans associate their deployment in Afghanistan with fun, given the enormous human costs of the war and the widespread recognition that the International Security Assistance Force’s (ISAF's) mission failed to bring peace and stability to the country?^71^

Returning to Fincham’s ‘peacetime’ model of fun helps to understand this puzzle. As described, Fincham defines fun as a fleeting and frivolous experience which, *like play*, often has a clear beginning and end. He further proposes that fun is experienced as a distraction from everyday problems and routines and involves at least a temporary alleviation of personal commitments and responsibilities. This is quite close to how interviewees described their deployment in Afghanistan and is captured by their common expression of ‘playing war’. As problematic as it might sound, this expression depicts many soldiers’ personal relationships to the war: a chapter in their life and story to tell; an opportunity to travel and explore the world; a chance to test and affirm their soldiering skills and experience something special and different from their peers at home; a temporary distraction from their domestic routines and responsibilities; and, above all, *not an existential war* nor a war where they felt a strong sense of commitment or responsibility to the civilian population but a *war of choice*, where personal risks were limited and calculated and winning the war was not their central objective nor a necessary condition for soldiers to feel good about their experiences.

Experiencing and representing war in this way rests upon and reinforces the image of Afghanistan as a playground for Western powers and their soldiers. Furthermore, seeing war this way erases Afghans’ everyday experiences as resilient subjects of serial war and violence.^72^ It is important, however, to emphasise that the expression ‘playing war’ was often used self-reflexively by interviewees to specify that they had not participated in a ‘real war’ with high risks and stakes, such as the current war in Ukraine or the war in Afghanistan seen from the perspective of the local population. Often interviewees were also critical of Norwegian politicians and military leaders, whom they accused of being unwilling to risk soldiers’ lives or give them sufficient resources to make a tangible difference. Soldiers’ representation of the war in Afghanistan as play was therefore not only or primarily an expression of colonial adventurism and disregard. In my interpretation, it was also a self-reflexive critique of their limited contributions, risks, and experiences as foreign soldiers in a professional expeditionary force with a ‘vague mandate to plant the Norwegian flag’, as one of them put it.

Interviewees also experienced soldiering as transgressive, though this argument requires some specification. As mentioned, Fincham suggests that the only type of fun people can have at work is the kind of fun that challenges the ideal of a good worker and institutional control of time and behaviour. Yet, as we have seen, interviewees described having fun while following orders and mastering their profession. Nevertheless, being a soldier at war was arguably experienced as transgressive in itself. As one of my interviewees reasoned:

our job as professional soldiers is really an ethical paradox, because at the end of the day, it is about using violence and sometimes even taking someone’s life, which goes against all the values we learned as children in school and church.

At the same time, I propose that soldiers’ sense of innocence was more important for their experience of fun in Afghanistan than their perception of soldiering as transgressive. As mentioned, Norwegian politicians and military leaders tend to represent Norwegian soldiers as exceptionally good and ethical, whether they are framed as peacebuilders or warriors. This Norwegian exceptionalism was often reproduced in interviews – even by soldiers who were critical of the framing of Afghanistan as a humanitarian intervention and what many described as ‘Norwegian naivety’. Whether as ‘innocent’ Norwegian citizens or apolitical soldiers, they also felt little responsibility for the causes and effects of the war they were fighting.^73^

## Conclusion: no longer fun?

To conclude, it bears mentioning that soldiers’ experiences of fun in Afghanistan were not only contextual but also contingent as they changed in response to time and events. For instance, some interviewees said they stopped having fun when the security situation in northern Afghanistan worsened and they were increasingly exposed to ambushes and IED attacks.^74^ The two IED attacks that killed five experienced soldiers in 2010 were particularly unsettling for interviewees who lost dear friends and colleagues. For some, these or other threatening episodes meant that sensations such as fear and grief became dominant –at least temporarily. Others, such as Katrine, began to question the meaning and value of the mission in Afghanistan seriously and concluded that it was not worth losing their life in this war. To stick with Fincham’s theory, we might say that the war was no longer experienced as play or could no longer be defined by an absence of seriousness.

Several interviewees also said they stopped having fun in Afghanistan when they gained increasing responsibilities. This could be either as leaders for troops of younger soldiers, or – as in Peter’s case – as parents with young children at home. Again, this corresponds with Fincham’s claim that responsibility is not a concern during periods of fun. Soldiering though is rarely as carefree and frivolous as Fincham’s examples from civilian life. Conversely, fun in war might depend on limited responsibilities and gravity rather than pure absence.

Finally, some said they no longer experienced war as fun after becoming older and more experienced, thus reaffirming the link between childishness and fun. No longer in their ‘carefree twenties’, they said they had ‘realised that they were not immortal’. Moreover, having ‘been there, done that’, the things they thought were fun as rookies no longer gave them the same sensations. One interviewee said he experienced ‘substitute fun’ when witnessing younger soldiers on their first mission. Following Fincham,^75^ I suggest that these interviewees accentuated their distance from an earlier stage of life, where they described themselves as young, naïve, and ignorant. They also created coherence between this life stage and their sense of self today as seasoned soldiers or mature and reflective civilians.

For a few soldiers, becoming older and ‘more mature’ also involved a loss of innocence, including the innocence of ‘not knowing better’. I illustrate this reasoning with a quote from my interview with Simon:

**Me:** Would you be interested in going back to Afghanistan on a new tour?

**Simon:** Yes and no. Yes, because I had such a good experience, I really did. But no, because I am a proper adult now and know better. I simply don’t have the conscience to shoot anyone there – not even a Taliban soldier who shoots at me – because I know now that he is probably just a poor farmer who needs money or is forced to participate in a war he does not want. There are just too many innocent people who have died in that war.

As Gloria Wekker observes about the Netherlands, expressions of national innocence rely on attachments to a self-image of being a small, tolerant, and ethical nation and a victim rather than a perpetrator of violence.^76^ This is also the case with Norway.^77^ Wekker also identifies a broader category of ‘white innocence’, which she describes as a way of being-in-the-world defined by ‘not-knowing but also not wanting to know’.^78^ In interviews, some veterans began to challenge both these forms of innocence by questioning the ethics and politics of Norway’s mission to Afghanistan and asserting more knowledge and understanding. In doing so, they assumed moral and personal responsibility beyond merely following the rules of war and military codes of conduct. As illustrated by Simon’s reply, they also redirected innocence to those who hardly experience the serial war in Afghanistan as fun: the civilian population.

## Footnotes

1 Meredith Lair, *Armed with Abundance: Consumerism and Soldiering in the Vietnam War* (Chapel Hill: University of California Press, 2011); Thomas Randrup Pedersen, ‘Ambivalent Anticipations: On Soldierly Becomings in the Desert of the Real’, *Cambridge Journal of Anthropology* 37, no. 1 (2019), 77–92.

2 Ben Fincham, *The Sociology of Fun* (London: Palgrave Macmillan, 2016), 1–2.

3 Ibid., 5.

4 Martha Wolfenstein, ‘The Emergence of Fun Morality’, *Journal of Social Issues* 7, no. 4 (1951), 15–25.

5 Walter Podilchak, ‘Distinctions between Fun, Leisure and Enjoyment’, *Leisure Studies* 10, no. 2 (1991), 133–48.

6 Mark Blythe and Marc Hassenzahl, ‘The Semantics of Fun: Differentiating Enjoyable Experiences’, in *Funology: From Usability to Enjoyment,* edited by Mark Blythe et al. (London: Kluwer, 2004), 91–100.

7 Fincham, 20–1.

8 Ibid., 41.

9 Ibid., 36.

10 Ibid., 203.

11 Ibid., 37.

12 Ibid., 170–5.

13 Ibid., 37, 200.

14 Ibid., 44.

15 Ibid., 204–5.

16 Ibid., 192.

17 Ibid., 202–4.

18 Ibid., 34.

19 Ibid., 197.

20 Ibid., 184.

21 Ibid., 187–200.

22 Ibid., 6.

23 Ibid., 41.

24 Ibid., 192.

25 Antonio De Lauri, ‘Introduction’, in *Antropologia*, edited by Antonio De Lauri (Milan: Ledizioni, 2013), 7–24.

26 Letter to Ivan Kashkin, 23 March 1939, Ernest Hemingway, *Ernest Hemingway Selected Letters 1917–1961*, edited by Carlos Baker (New York: Scribner Book Company, 2009 [1981]), 480.

27 Zöe H. Wool, *After War: The Weight of Life at Walter Reed* (Durham, NC: Duke University Press, 2015).

28 Pedersen.

29 Kenneth MacLeish, ‘How to Feel about War: On Soldier Psyches, Military Biopolitics, and American Empire,’ *BioSocieties* 14, no. 2 (2019), 274–99.

30 Eva Johais, ‘The WARFUN Taboo’, *War & Society* 44, no. 1 (2025).

31 Marianne Gullestad, *Plausible Prejudice* (Oslo: Universitetsforlaget, 2006); Heidi Mogstad, *Humanitarian Shame and Redemption: Norwegian Citizens Helping Refugees in Greece* (New York: Berghahn Books, 2023).

32 Halvard Leira, ‘“Our Entire People Are Natural Born Friends of Peace”: The Norwegian Foreign Policy of Peace,’ *Swiss Political Science Review* 19, no. 3 (2013), 338–56.

33 Synne L. Dyvik, ‘“Valhalla Rising”: Gender, Embodiment and Experience in Military Memoirs’, *Security Dialogue* 47, no. 2 (2016), 133–50.

34 Harald Høiback, *Forsvaret: Et Kritisk Blikk fra Innsiden* (Oslo: Cappelen Damm, 2016).

35 Anders Hammer, *Faryab – Arven etter Norge* (Oslo: Press, 2019).

36 Victoria Basham, ‘Waiting for War: Soldiering, Temporality and Gendered Politics of Boredom and Joy in Military Spaces,’ in *Emotion, Politics and War*, ed. by Linda Åhäll and Thomas Gregory (London: Routledge), 128–40.

37 Lair.

38 Katherine E. Brown and Elina Penttinen, ‘A “Sucking Chest Wound” Is Nature’s Way of Telling You to Slow Down …’, *Critical Studies on Security* 1, no. 1 (2013), 124–26; John Hockey, *Squaddies: Portrait of a Subculture* (Exeter: Exeter University Publications, 1986).

39 De Lauri.

40 John T. Parry, ‘“Just for Fun”: Understanding Torture and Understanding Abu Ghraib’, *Journal of National Security Law and Policy* 1 (2005), 253–84.

41 Ibid., 281.

42 Carsten Jensen, *Den Første Sten*, trans. Håvard Syvertsen (Oslo: Press, 2017).

43 Ibid., 450.

44 Norwegian Armed Forces, ‘Afghanistan’ (2021), <https://www.forsvaret.no/om-forsvaret/operasjoner-og-ovelser/internasjonale-operasjoner/afghanistan> [accessed 15 June 2023].

45 Nina Græger and Halvard Leira, ‘Norwegian Strategic Culture after World War II: From a Local to a Global Perspective’, *Cooperation and Conflict: Journal of the Nordic International Studies Association* 40, no. 1 (2005), 45–66.

46 Utenriksdepartementet og Forsvarsdepartementet, *‘En god alliert – Norge i Afghanistan 2011–2014’*, NOU 2016, no. 8 (Oslo: Departementenes servicesenter, Informasjonsforvaltning), <https://www.regjeringen.no/contentassets/09faceca099c4b8bac85ca8495e12d2d/no/pdfs/nou201620160008000dddpdfs.pdf> [accessed 15 June 2023].

47 Hammer.

48 ‘Afghanistan Before and After 20 Years of War (2001–2021)’, <https://watson.brown.edu/costsofwar/Afghanistanbeforeandafter20yearsofwar> [accessed 15 June 2023].

49 Gudmund Waaler, Gerry Larsson and Sofia Nilsson, *Voldsbruk og Livsfare: Norske og Svenske Soldaters Krigserfaringer fra Afghanistan* (Oslo: Cappelen Damm, 2019).

50 Kristian B. Harpviken, ‘Dømt til å Mislykkes? USA og det Afghanske Statsbyggingsprosjektet, 2001–2021’, *Internasjonal Politikk* 80, no. 2 (2022), 257–70.

51 Græger and Leira.

52 A few interviewees with long careers in the army said they had been ‘ordered’ to Afghanistan. Nonetheless, these individuals emphasised that serving abroad was ‘part of their job’, ‘important for their careers’, and something they ‘wanted to experience’. For a more detailed discussion of my interviewees' desires to experience war and combat in Afghanistan see: Heidi Mogstad. 'The self-realising soldier: Post-heroic reflections from Norwegian Afghanistan veterans', *Public Anthropologist* 6, no. 1 (2024), 79–104.

53 Hans J. Bøe et al., *Afghanistanundersøkelsen 2020: Veteraners Erfaringer, Helse og Livskvalitet etter Tjeneste i Afghanistan 2001–2020* (Oslo: Forsvaret, 2021).

54 Nick Caddick, Alex Cooper, and Brett Smith, ‘Reflections on Being a Civilian Researcher in an Ex-Military World: Expanding Horizons?’, *Critical Military Studies* 5, no. 2 (2019), 95–114.

55 Fincham.

56 Caddick, Cooper, and Smith.

57 Sebastian Mohr, Birgitte R. Sørensen, and Matti Weisdorf, ‘The Ethnography of Things Military – Empathy and Critique in Military Anthroplogy’, *Ethnos* 86, no. 4 (2021), 600–15.

58 Lair.

59 Bård Mæland and Paul Otto Brunstad, *Enduring Military Boredom: From 1750 to the Present* (Houndmills: Palgrave Macmillan, 2009).

60 Fincham.

61 Yuval N. Harari, ‘Armchairs, Coffee and Authority: Eye-Witnesses and Flesh Witnesses Speak about War, 1100–2000’, *The Journal of Military History* 74, no. 1 (2010), 53–78.

62 Veena Das, *Life and Words: Violence and the Descent into the Ordinary* (Berkeley and Los Angeles: University of California Press, 2006).

63 Cynthia Enloe, *Does Khaki Become You? The Militarization of Women’s Lives* (London: Pluto, 1983).

64 Basham.

65 Tone Danielsen, *Making Warriors in a Global Era: An Ethnographic Study of the Norwegian Naval Special Operations Commando* (Lanham, MD: Lexington Books, 2018); Ole M. Totland, ‘Det Operative Felleskapet. En Sosialantroplogisk Studie av Kropp, Kjønn og Identitet blant Norske Soldater i Telemark Bataljon’ (Master’s thesis, Institute for Social Anthroplogy, University of Oslo, 2009).

66 Erin P. Finley, *Fields of Combat: Understanding PTSD among Veterans of Iraq and Afghanistan* (Ithaca, NY: ILR Books, 2011); Wool.

67 Grégorie Hervouet-Zeiber, ‘The “Salt” of Life: Of Wars and Their Domesticities in Contemporary Russia’, *American Ethnologist* 50 (2023), 43–53.

68 Dyvik, 143.

69 Julia Welland, ‘Joy and War: Reading Pleasure in Wartime Experiences,’ *Review of International Studies* 44, no. 3 (2018), 438–55.

70 Steinar Aas, ‘The Role of the Polar Explorer in the Norwegian Identity,’ in *I viaggi e la modernita. Dalle grandi esplorazioni geografiche ai mondi extraterrestri,* ed. by Annalisa D’Ascenzo (Rome: CISGE – Centro Italiano per gli Studi Storico-Geografici, 2021), 235–48.

71 Harpviken.

72 Anila Daulatzai, ‘Remaining Undone: Heroin in the Time of Serial War,’ in *War and Health: The Medical Consequences of the Wars in Iraq and Afghanistan*, ed. by Catherine Lutz and Andrea Mazzarino (New York: New York University Press, 2019), 73–89.

73 Waaler, Larsson og Nilsson.

74 Hammer.

75 Fincham, 188.

76 Gloria Wekker, *White Innocence: Paradoxes of Colonialism and Race* (Durham, NC: Duke University Press, 2016).

77 Gullestad.

78 Wekker, 17.

